# Sensors in a Flash! Oxygen Nanosensors for Microbial Metabolic Monitoring Synthesized by Flash Nanoprecipitation

**DOI:** 10.1021/acssensors.2c00859

**Published:** 2022-09-02

**Authors:** Tony Tien, Samuel C. Saccomano, Pilar A. Martin, Madeleine S. Armstrong, Robert K. Prud’homme, Kevin J. Cash

**Affiliations:** † Chemical and Biological Engineering, Colorado School of Mines, Golden, Colorado 80401, United States; ‡ Chemical and Biological Engineering, Princeton University, Princeton, New Jersey 08544, United States; § Quantitative Biosciences and Engineering, Colorado School of Mines, Golden, Colorado 80401, United States

**Keywords:** Nanoparticle fabrication, nanosensors, flash nanoprecipitation, FNP, oxygen, metabolism

## Abstract

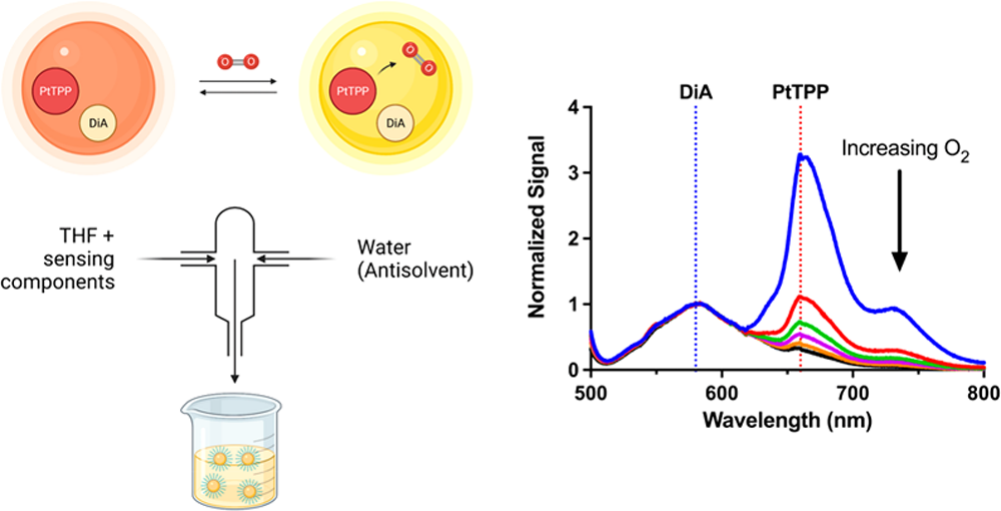

Flash nanoprecipitation (FNP) is an efficient and scalable nanoparticle synthesis method that has not previously been applied to nanosensor fabrication. Current nanosensor fabrication methods have traditionally exhibited poor replicability and consistency resulting in high batch-to-batch variability, highlighting the need for a more tunable and efficient method such as FNP. We used FNP to fabricate nanosensors to sense oxygen based on an oxygen-sensitive dye and a reference dye, as a tool for measuring microbial metabolism. We used fluorescence spectroscopy to optimize nanosensor formulations, calibrate the nanosensors for oxygen concentration determination, and measure oxygen concentrations through oxygen-sensitive dye luminescence. FNP provides an effective platform for making sensors capable of responding to oxygen concentration in gas-bubbled solutions as well as in microbial environments. The environments we tested the sensors in are*Pseudomonas aeruginosa* biofilms and*Saccharomyces cerevisiae* liquid cultures—both settings where oxygen concentration is highly dependent on microbial activity. With FNP now applied to nanosensor fabrication, future nanosensor applications can take advantage of improved product quality through better replicability and consistency while maintaining the original function of the nanosensor.

Flash nanoprecipitation (FNP) is a technique for the efficient and consistent fabrication of polymer-based nanoparticles. FNP is a highly tunable and scalable formulation method that has produced nanoparticles from the mg to ton scale.^1^ The process takes advantage of a rapid mixing process to create a supersaturated solution of nanoparticle components, which causes the precipitation of nanoparticles through the mixing of a solvent and antisolvent.^2^ Other nanoprecipitation techniques have been developed for the creation of nanoparticles, but issues with these approaches remain ever-present with inconsistent replicability, size uniformity, and product yield.^1,3^ During FNP, when the component mixing streams flow rapidly and turbulently, nanoparticle size and composition can be related directly to stream composition with little effect from streamflow rate, while the stability is affected by formulation choice rather than fabrication parameters.^4^ Through process and formulation tuning, FNP can create nanoparticles with a controlled diameter from approximately 40 to 400 nm—useful in applications where particle size is related to function, such as biocompatible protein conjugation and biodistribution of the particles.^5,6^ With this approach, researchers have used FNP for applications such as drug delivery^7−10^ and optical imaging applications.^10−12^

To date, FNP has not been applied for the fabrication of nanosensors. Nanosensors change their optical properties in response to analyte concentration changes—enabling optical measurement of local conditions. Optical polymeric nanosensors are particularly well suited for the detection of analytes due to their small size and tunability of the response based on formulation.^13,14^ Nanosensors are particularly of interest for use *in situ* due to their small sizes, inert and nontoxic formulations, and potential to function in biologically relevant pH ranges.^15^ Additionally, nanoparticles provide a unique application for imaging as they can be used to measure 3-dimensional spatial gradients and temporal changes in complex, heterogeneous samples.^16^ However, the current fabrication approach for nanosensors is reliant on emulsification solvent evaporation (ESE)—yielding minimal nanoparticle size control, large polydispersity, and poor options to select specific nanoparticle sizes while retaining sensor function.^17^ While some alternate approaches with different materials have shown some limited size tuning,^18^ the current approaches do not offer the control over nanoparticle fabrication seen with FNP.

While there are many approaches to designing quantitative optical sensors, one common approach is to measure ratiometric luminescence changes. In these approaches, there are two luminescent signals from the sensor based on two separate dyes: one which changes with analyte concentration, and one which does not change with analyte concentration to be used as a reference, as shown in Figure 1. For more detail on ratiometric approaches, please see Doussineau et al.^19^ The incorporation of these sensing components into a polymeric matrix creates what we call a nanosensor. The ratio of these two dyes remains fixed once encapsulated, which enables analyte measurements in complex biological systems by adjusting for nanoparticle concentration.^16,20,21^ Nanosensors have been developed for a variety of biologically relevant parameters such as pH,^22^ electrolytes,^23^ glucose,^24^ heavy metal ions,^25^ and pesticides.^26^ A wide variety of analyte nanosensors can be made by simply changing sensing components while tuning the formulation for the concentration range of interest. Consequently, improving the fabrication method of nanosensors has the potential to bolster nanosensor resilience and consistency for use across the wide range of existing applications.

**Figure 1:**
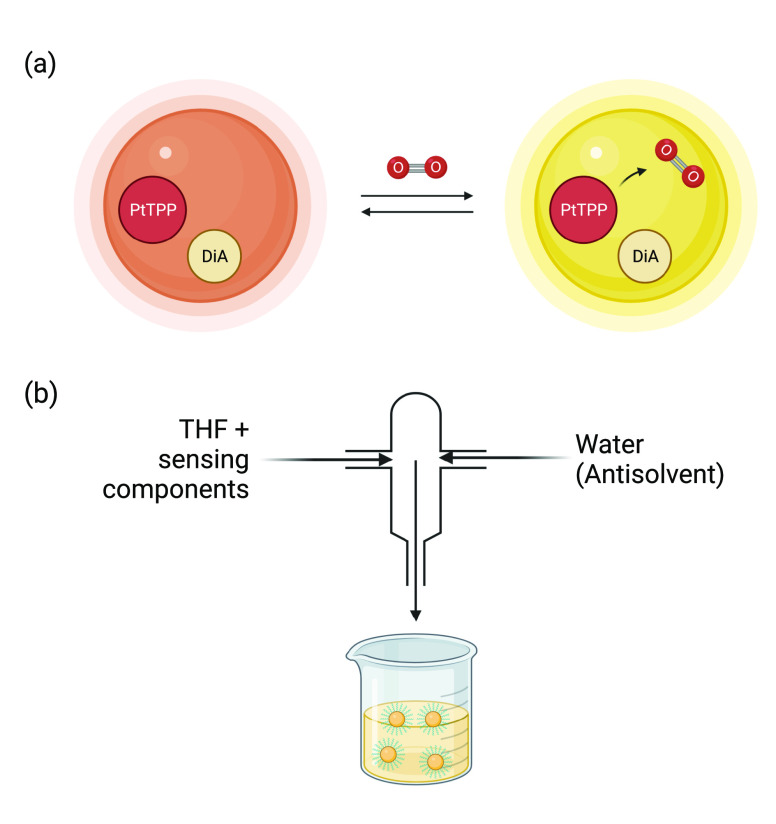
We fabricated oxygen-sensitive nanosensors using flash nanoprecipitation (FNP) with sensing components used in other oxygen nanosensors. (a) The oxygen sensing mechanism of the sensors is based on PtTPP—a dye which luminesces most brightly in the absence of oxygen and decreases in luminescence with increasing oxygen concentrations. DiA, used as a reference dye, luminesces at a consistent level regardless of oxygen concentration. The presence of DiA as a reference dye allows for oxygen measurements, which are independent of many artifacts (e.g., sensor concentration). (b) A confined-impinging jet mixer (CIJ) is used to turbulently mix oxygen sensing components in THF with water as an antisolvent, resulting in nanosensors fabricated with FNP. Created with BioRender.com.

Given that oxygen-sensing dyes have already been well characterized in nanosensors,^16,27,28^ oxygen sensors make for an ideal system to study the FNP formulation method. The biological relevance of these sensors would benefit from improved replicability with a successful implementation in FNP.

Nanoscale systems for measuring biological samples have become increasingly important due to the need for methods which can capture spatial and temporal dynamics. The metabolic activity of a microbial community is important to understand the vitality and viability of the consortia in response to the environmental conditions.^29^ Under favorable growth conditions, such as high nutrient availability, cell metabolism is more likely to be high while unfavorable conditions are more likely to slow or cease metabolic activity. For example, metabolic activity has become a common proxy for determining antibiotic susceptibility of antimicrobial resistant species.^20,30,31^ Additionally, metabolic activity has a widespread effect on the surrounding as the depletion or production of metabolites can have a profound effect on community.^32^

Many systems have been used to sense a variety of indicators related to metabolic activity. Most of these sensors target key biomolecules which are either consumed or produced by the microbial community of interest. Yeor-Davidi et al. used silicon nanowires to detect redox active metabolites such as glucose which could be oxidized by oxidase enzymes. As metabolites are oxidized hydrogen peroxide is formed and detected through a change in electrochemical potential.^33^ Progress has been made to monitor NADP and NADPH levels in biological systems through fluorescent probes. Goldbeck et al. used a blue fluorescent protein (mBFP) to track NADPH levels in *Corynebacterium glutamicum* and *Escherichia coli*.^34^ Surger et al. used an optical gas-phase carbon dioxide sensor for the detection of microbial growth of multiple species in contaminated heating oils and diesel fuels.^35^ A variety of other methods have been discussed by Braissant et al. which include the detection of ATP, stable isotopic labeling, isothermal calorimetry, and the monitoring of various electron acceptors such as nitrate, sulfate, metal ions, and oxygen.^29^

Oxygen is one of the most common indicators of metabolic activity because of its role in aerobic respiration. Rapid oxygen consumption within a system is evidence of a thriving microbial community. Oxygen monitoring methods are most commonly categorized into electrochemical methods, optode films, and nanoparticle/microparticle sensors. The clark electrode is one of the most common methods of oxygen detection due to its high sensitivity; however, oxygen is consumed in the process and this method is often disruptive to the system under investigation.^36,37^ Optode films are advantageous for measuring microbial growth on a surface; however, due to geometric limitations optode films tend to have slower response times and are limited to 2-dimensional imaging applications.^38^ Particle-based approaches are ideal for oxygen monitoring due their fast response and uniform dispersity within the system, allowing them to capture spatial and temporal dynamics.

In this work, we show that FNP provides a platform for easily fabricating nanosensors and can be controlled to produce nanosensors of replicable size and composition. We demonstrate FNP’s potential with nanosensors in measuring oxygen concentrations, showing that FNP-based nanosensors respond similarly to prior oxygen sensors and function well in complex biological settings. FNP is a new approach to fabricate polymer-based nanosensors, opening the nanosensor field for the future potential of producing nanosensors with lower barriers of accessibility and new synthesis materials that have yet to be explored.

## Experimental Section

### Methods

For nanosensor fabrication, platinum(II) 5,10,15,20-(tetraphenyl)porphyrin (PtTPP) was purchased from Frontier Scientific (Logan, UT, USA), 4-(4-dihexadecylaminostyryl)-*N*-methylpyridinium iodide) (DiA) was purchased from ThermoFisher Scientific (Waltham, MA, USA), and polystyrene-*block*-polyethylene glycol (PS–PEG) was from Polymer Source, Inc. (Montreal, QC, CA); Vitamin E, Vitamin E acetate, and tetrahydrofuran were purchased from MilliporeSigma (St. Louis, MO, USA).

For glucose oxidase assays, α-d-Glucose, Glucose Oxidase from *Aspergillus niger*, and Dulbecco’s Phosphate Buffered Saline (dry powder, DPBS Modified, without calcium, without magnesium, suitable for cell culture) were purchased from MilliporeSigma (St. Louis, MO, USA). 96-well black-walled optical bottom plates from ThermoFisher Scientific (Waltham, MA, USA) were used to contain the samples.

For pH testing, a Fisherbrand accumet AB150 pH meter and probe, 10 N HCl, and 10 N NaOH from ThermoFisher Scientific (Waltham, MA, USA) were used.

For antibiotic susceptibility testing, *Pseudomonas aeruginosa* PAO1 was purchased from ATCC (Manassas, VA, USA), colistin sulfate was purchased from MilliporeSigma (St. Louis, MO, USA), *Saccharomyces cerevisiae* (brewing yeast) strains MIP-510 (Kolsch I) and MIP-354 (Kveik: Oslo) were purchased from Propagate Laboratories (Golden, CO, USA), and Campden tablets (potassium metabisulfite, PMB) obtained from Crosby and Baker (Westport, MA, USA). 96-well black-walled optical bottom plates from ThermoFisher Scientific (Waltham, MA, USA) were used to contain the samples.

For the gas bubbling setup, ultra-high-purity nitrogen gas and compressed air from Matheson (Denver, CO, USA), a 10 mm path length quartz cuvette with rubber septa seal from Starna Cells (Atascadero, CA, USA), Aalborg nitrogen mass flow controller (Orangeburg, NY, USA), and an air mass flow controller from Alicat Scientific (Tucson, AZ, USA) were used.

### Nanosensor Fabrication

Nanosensors were produced via FNP using a confined-impinging jet mixer (CIJ) as described previously and as shown in Figure 1b.^30^ Briefly, PtTPP, DiA, PS–PEG, and either Vitamin E or Vitamin E acetate were dissolved in a THF stream and rapidly mixed against a DI water antisolvent stream into a quench bath of DI water to drive controlled precipitation and produce the stabilized nanosensors. Nanosensor size was measured by dynamic light scattering (Malvern Nanosizer, Westborough, MA, USA). The concentrations of components in the THF feed stream were adjusted to obtain nanosensors with different compositions and explore the effect of various parameters on the nanosensors’ performance. The varied parameters include the ratio of the oxygen-responsive dye, PtTPP, to the reference dye, DiA, the total mass concentration of the core of the nanosensor, the concentration of the PS–PEG stabilizer, and the use of either Vitamin E or Vitamin E acetate as the main core component. The compositions are given in Table 1. Emulsification solvent evaporation sensor fabrication was adapted from Jewell et al.^16^ The dyes were replaced with 1.25 mg of PtTPP and 0.5 mg DiA in the optode formulations.

**Table 1:** Formulations of FNP Oxygen Nanosensors Tested

Sample Number	PtTPP:DiA (mg:mg)	PS–PEG (mg/mL)	Core (mg/mL)	Core Component
1	5:0.2	5	5	Vitamin E acetate
2	5:0.2	1.25	5	Vitamin E acetate
3	5:0.2	5	5	Vitamin E
4	5:0.2	1.25	5	Vitamin E
5	5:2	5	5	Vitamin E acetate
6	5:2	1.25	5	Vitamin E acetate
7	5:0.2	5	10	Vitamin E acetate
8	5:0.2	1.25	10	Vitamin E acetate
9	5:2	5	10	Vitamin E acetate
10	5:2	1.25	10	Vitamin E acetate

### Sample Selection via Glucose Oxidase Assay

Ten variations of FNP nanosensors (Table 1) were tested on a BioTek Synergy H1 Hybrid Multi-Mode microplate reader (Winooski, VT, USA). After samples were added to a 96-well optical bottom well plate, they were excited at 488 nm to generate luminescence spectra in intervals of 1 nm, and ratiometric data was analyzed to find the best sample. Optimal samples had similar magnitudes of PtTPP and DiA luminescence, greater signal intensity, and greater signal contrast between deoxygenated and oxygenated states. A glucose oxidase assay was performed using 100 mM glucose and 20 IU/mL glucose oxidase stocks in PBS. Each well tested contained 60 μL of nanosensors. Deoxygenated samples contained 18 μL each of the glucose and glucose oxidase stocks with an additional 84 μL of PBS. Oxygenated controls contained PBS only, glucose and PBS, or glucose oxidase and PBS with the same total well volume of 180 μL.

### Calibration Curves and Reversibility

The FNP oxygen nanosensors were tested for response to changes in oxygen concentration in an air-nitrogen gas bubbling system as described by Saccomano et al.^21^ Oxygen calibration curves and reversibility tests were completed with an Avaspec 2408L spectrometer (Avantes Inc., Louisville, CO) and analyzed using Avantes AvaSoft 8. Two mL of nanosensors were used in a septum sealed quartz cuvette and fitted with gas line and vent using 22-gauge needles. Gas flow rates from an air and nitrogen tank were controlled by mass flow controllers and mixed in a 25 mL mixing chamber to form gas streams with various mole fractions of oxygen from 0% to 21% or 0 to 6.65 mg/L dissolved oxygen at 5,780 ft elevation (Golden, CO). Gas was bubbled into the nanosensor solution in the cuvette at a total flow rate of 20 mL/min. To test the luminescence at each dissolved oxygen concentration the gas stream was allowed to bubble for 20 min at which point the needle was removed and a fiber optic 532 nm LED was shone on the cuvette. This was repeated for 0 to 6.65 mg/L dissolved oxygen concentrations in increments of 0.79 mg/L (2.5%). Sensor reversibility was tested by alternating between 0 mg/L dissolved oxygen (nitrogen bubbling) and 6.65 mg/L dissolved oxygen (air bubbling) at 20 min each to observe the response of the sensors to quenched and unquenched states. The samples were measured over three cycles of oxygenation and deoxygenation states.

### Pseudo-Stern–Volmer Analysis

The calibration curve data was used to perform a pseudo-Stern–Volmer analysis. To measure the luminescence of the individual dyes, the maximum peak intensities at 580 nm (DiA) and 660 nm (PtTPP) were taken. The ratiometric signal was determined by dividing the luminescence of the of the oxygen peak (PtTPP) to the reference peak (DiA). A pseudo-Stern–Volmer plot was generated by dividing the ratiometric signal in the absence of oxygen by the ratiometric signal at each of the tested oxygen concentrations using the Stern–Volmer equation. These values are plotted with respect to oxygen concentration, generating a linear correlation between the luminescence ratio and oxygen concentration. A linear regression of the plot was taken to determine the pseudo-Stern–Volmer constant *K*_pSV_.

### Functional Lifetime

We tested the viability of the sensor response over an extended period of time with expected sensor and dye degradation over time. The sensors were evaluated at 40 days and 100 days from sensor fabrication using the glucose oxidase assay as described above.

### pH Response

PBS samples were prepared in a pH range of 5–8 in intervals of 1, as well as a stock pH of 7.40. The pH of the various PBS samples was adjusted using 1 N HCl and 1 N NaOH (diluted from their respective 10 N stocks) using live measurements with a pH electrode. 160 μL of nanosensors were mixed with 40 μL of each PBS variation in a 96-well plate. The samples were excited at 488 nm in intervals of 1 nm to generate emission spectra from 500 to 700 nm analyze dye signal changes in an ambient oxygen environment.

### Antibiotic Susceptibility Test (AST) with *Pseudomonas aeruginosa*

Nanosensors were concentrated to 10× using MilliporeSigma Amicon Ultra-0.5 Centrifugal Filter Units (St. Louis, MO, USA) and a microcentrifuge. Setup and execution of the AST was adapted from Jewell et al.^20^ The antibiotic challenge plate was set up as follows: column 1 contained no antibiotic, column 2 was blank, and columns 3–10 contained serial half dilutions of 250 μg/mL colistin sulfate in PBS. Columns 11–12 contained PBS to observe the growth of the biofilm in absence of nanosensors.

### Plate Reader Analysis for AST

Luminescence end point readings were taken at 488 nm excitation. Emission wavelengths of 580 and 660 nm were used for DiA and PtTPP, respectively. The ratiometric signal of the oxygenated peak (PtTPP) to deoxygenated peak (DiA) was compared to antibiotic concentration. The luminescence readings were normalized to the blank readings, after which we fitted a linear regression to each column’s data from 0 to 5 h. The slope of the ratiometric signal was graphed as a function of antibiotic concentration.

### Tracking Metabolic Changes with *Saccharomyces cerevisiae* (Brewing Yeast) Strains MIP-510 (Kolsch I) and MIP-354 (Kveik:Oslo)

Metabolic activity was measured with the nanosensors using a protocol similar to that in Saccomano et al.^21^ The yeast strains were diluted in their respective wort solutions at 1:3, 1:10, and 1:100 concentrations. Samples were dispensed into a 96-well plate with all permutations of the given yeast strains, yeast dilutions, and potassium metabisulfite (PMB) condition in quadruplicate. In addition, controls of each strain without nanosensors and nanosensors with/without PMB were performed in sextuplicate. The samples were incubated at 30 °C and shaken for 5 min before each reading, with readings taken every 15 min over a 60 h period. Aluminum foil was used to cover the 96-well plate with minute holes punched into the foil for potassium metabisulfite addition at 42 h.

## Results

Our nanosensors contain two dyes, PtTPP and DiA, which are used to sense changes in oxygen concentration. DiA was chosen as the oxygen-insensitive reference dye, and PtTPP was chosen as the oxygen-responsive dye. Oxygen functions as a quenching agent for PtTPP, where PtTPP luminescence decreases with increasing oxygen concentrations. At low oxygen concentrations, luminescence quenching is minimized resulting in greater signal from the PtTPP dye. As oxygen concentrations increase, it quenches only the PtTPP, leaving the DiA luminescence unchanged, which changes the overall luminescence ratio between the two dyes.

In fabricating nanosensors using FNP, the formulation of the supporting matrix is different from our prior sensors. These FNP nanosensors contain PS–PEG and a core material of either Vitamin E or Vitamin E acetate. However, this change in material properties is not a concern for the sensing mechanism, as other work has already demonstrated that for this class of sensors, the structural nanoparticle matrix is easily adaptable to other materials assuming the hydrophobicity remains similar, a result extended in this work.^14,18,39^

The first step in the process of developing these new FNP produced nanosensors was to determine the impact of formulation parameters on the nanosensor response. The key parameters we tested were as follows: oxygen-responsive dye to reference dye ratio (PtTPP:DiA), structural component amounts (PS–PEG, Core), and core component identity (vitamin E or vitamin E acetate), as seen in Table 1. The designations in mg/mL in the table represent the concentrations of the components in the solvent stream introduced in the CIJ mixer. Each sample was measured to compare particle size and size distribution as seen in Supporting Information (SI) Figures S1 and S2. Greater average particle diameter was correlated to smaller PS–PEG amounts and larger core amounts highlighting the ability to tune particle size. We also assessed luminescence output from the nanosensors and their response to oxygen concentration.

Resulting luminescence spectra for all formulations are shown in Figures S3 and S4 in SI. Formulations containing the 5:2 ratio of PtTPP:DiA provided reference dye signals (DiA) similar in magnitude to oxygen-responsive dye signals (PtTPP). This is desirable, as ratios of different magnitudes can complicate sensor interrogation with a single measurement. Nanosensor formulations containing a lower concentration of PS–PEG (1.25 mg/mL) exhibited ∼20–50% decreases in luminescence intensity compared to 5 mg/mL counterparts, creating a preference for higher PS–PEG concentrations. Higher quantities of core material (10 mg/mL) reduced the luminescence intensity of the reference dye (DiA). For this reason, lower amounts of core material are preferred to ensure similar peak sizes between the two dyes. Furthermore, Samples 3 and 4, which contained Vitamin E in place of Vitamin E acetate, exhibited lower overall luminescence when compared to the other samples. Given that absorbance data between the formulations tested were comparable in value, Vitamin E appears to adversely affect sensor luminescence, potentially through quenching of the nanosensor signal, since Vitamin E functions as an antioxidant. Therefore, we used formulations containing Vitamin E acetate to achieve greater signal intensities. Given the desirable characteristics and parameters from this screening, sample 5’s formulation (5:2 PtTPP:DiA; 5 mg/mL PS–PEG, 5 mg Vitamin E acetate) exhibited the optimal balance between ratiometric signal of the two dyes, luminescence signal intensity, and response to oxygen. This formulation was used for all the following characterization tests.

The encapsulation efficiency of the Flash Nanoprecipitation approach for the optimized sensor composition was measured by comparing the absorbance value of the dyes in the filtrate and the retentate of samples filtered through a 30k MW amicon column. No signal was found in the filtrate where any free dye molecules would be found indicating that close to 100% of the dye was in the particles (see Figure S5). We evaluated the loading efficiency of the FNP particles as compared to a standard emulsification solvent evaporation method by comparing the absorbance of the two dyes in solution of each sensor batch. Each method was loaded with equivalent masses of the two dyes per 5 mL batch, 125 μg of PtTPP and 50 μg of DiA. The FNP batch clearly showed a greater absorbance above the background than the ESE sensors (see Figure S5) which is supported by the observation that dye is observed in large aggregates left over from the sonication process, indicating that some amount of the dye did not make it into the particles. We verified that dye leaching from the particle was very slow based on filtering out any free dye through a 30k molecular weight filter and measuring the change in absorbance over time. Over 2 weeks we saw no increase in absorbance (Figure S5).

Aggregation caused quenching (ACQ) was looked into as a potential phenomenon which may be present when we encapsulate our dyes in nanoparticles. Figure S6 shows that fluorescence was brighter in the nanoparticle phase versus an organic solvent phase, indicating that ACQ is unlikely as we would expect the brightness to decrease relative to that in solution.

Sensors made by FNP and ESE methods were characterized by DLS to obtain a comparison of nanoparticle size and zeta potential between the two methods (Figure S7). We tested stability of the two methods using the sample 5 formulation as our test case. To measure stability of the nanoparticle itself we measured the size of the sensor batch at 0 weeks, 1 week, and 2 weeks from the initial synthesis. Results showed that there were no significant changes in particle size over that time period (see Figure S8).

**Figure 2:**
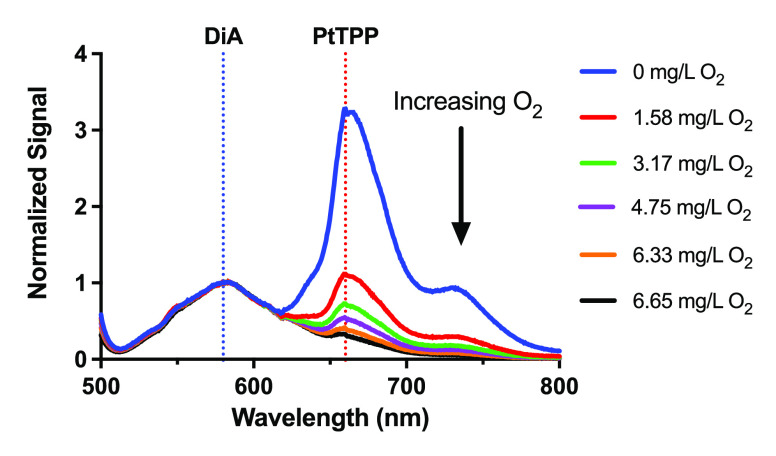
Normalized luminescence spectra for ratiometric nanosensors containing an oxygen-sensitive PtTPP dye and an oxygen-insensitive DiA dye at varying dissolved oxygen concentrations. Oxygen nanosensors change in luminescence according to ambient oxygen changes by bubbling air/nitrogen mixtures into the sample within a cuvette system. Dashed lines indicate the peak wavelengths for the reference dye (DiA) at 580 nm and the oxygen-responsive dye (PtTPP) at 660 nm. As oxygen concentration is increased from 0 mg/L to 6.65 mg/L, PtTPP luminescence is quenched, while DiA luminescence remains relatively constant. Data was normalized to peak intensity at 580 nm.

**Figure 3:**
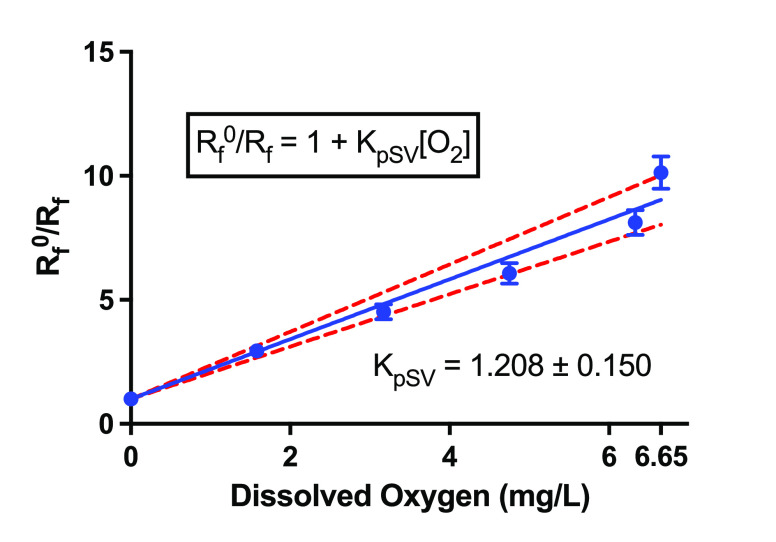
Pseudo-Stern–Volmer (pSV) plot of the ratiometric in response to oxygen concentration change from 0 mg/L to 6.65 mg/L, where 6.65 mg/L represents the dissolved oxygen mixture at 5780 ft elevation. A relatively linear slope indicates optimal ratiometric behavior of the sensor response, allowing for a pseudo-Stern–Volmer constant (*K*_pSV_) to be extrapolated to determine sensor response at oxygen concentrations not included in initial testing. The error bars represent standard deviation for *n* = 3 replicates. Dotted lines indicate 95% confidence interval of the linear fit.

When our nanosensor formulation was tested for oxygen response from 0 to 6.65 mg/L a notable response can be observed in the luminescence of PtTPP at 660 nm, which decreases as oxygen concentration increases (see Figure 2). Minor changes in the luminescence of the reference dye, DiA, can also be observed at 580 nm; the intensity of the DiA peak decreased with increasing oxygen concentration as well, as observed in Figure S9. To counteract this issue, a ratiometric approach was used to normalize the spectra to the DiA peak allowing us to better evaluate the oxygen response. The spectra shows a general decrease in signal ratio with increasing oxygen concentration, as shown in Figure 2. A pseudo-Stern–Volmer plot, as shown in Figure 3, demonstrates the relationship with changing oxygen concentration in terms of the change in luminescence ratio compared to that ratio at zero oxygen. Despite slight fluctuations in the luminescence of DiA, a linear relationship is still achieved over the full oxygen range, as shown in Figure S10.

When tested for reversibility, the luminescence ratio increased by 67.4% over three reversibility cycles for oxygenated environments and increased by 54.2% for deoxygenated environments (see Figure S11a). The increasing luminescence ratio over an increased number of reversibility cycles is driven by decreases in DiA signal over time with increasing PtTPP signal over time, as also observed in Figure S3b. When analyzed separately, PtTPP increases in deoxygenated luminescence by approximately 17.5% after three cycles, with the DiA signal being reduced by 39.0% in the same time frame (see Figure S11b,c).

The nanosensors were also periodically tested for signal stability over a period of four months. Between 40 and 100 days after fabrication, the reference dye (DiA) was less stable when treated with glucose and glucose oxidase, producing a noticeable signal drop from the oxygenated control (see Figure S12). Even though the overall signal of the sensors reduced by approximately 50%, the sensors remained responsive to oxygen—though with a different calibration curve—necessitating recalibration for quantitative application. The nanosensor response was tested in a variety of pH conditions from pH 5 to 8. The pH had little effect on the raw intensity or the response of the sensors to oxygen (see Figure S13).

**Figure 4:**
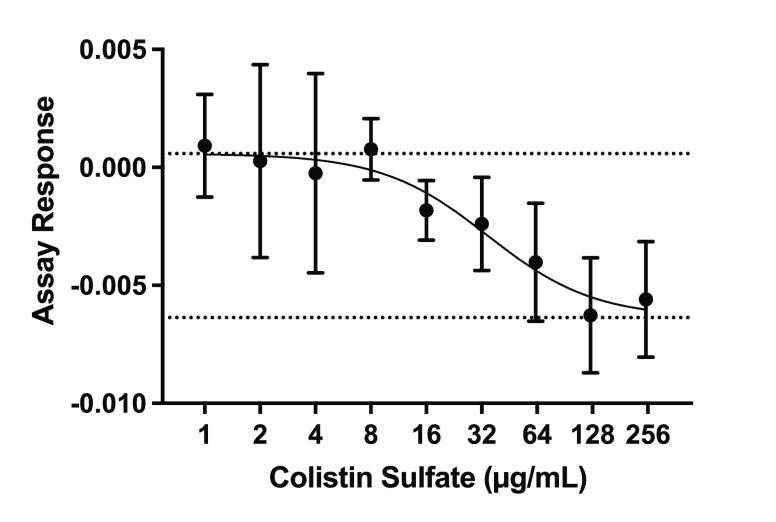
Metabolic response of *P. aeruginosa* biofilms with varying concentration of colistin sulfate as measured by oxygen nanosensors. Nanosensors were grown in *P. aeruginosaPAO1* biofilms for antibiotic susceptibility testing (AST). The assay response represents the slope of the ratiometric signal (oxygen consumption) within the biofilms from over the first 5 h of antibiotic exposure; higher values indicate greater oxygen consumption over time, whereas values close to 0 represent minimal oxygen consumption change over time. Error bars represent standard deviation of the assay response for *n* = 4 replicates. Raw luminescence plots for individual dyes and ratiometric signals are provided in Figures S14–S15 in SI.

To test the function of these nanosensors in biological systems, we applied them to monitor metabolic oxygen consumption in *Pseudomonas aeruginosa* biofilms in response to antibiotic concentration log(2) dilution series using a previously developed assay.^20^ Under natural conditions these biofilm-forming microbes rapidly deplete oxygen in the extracellular matrix; however, when exposed to high colistin sulfate (antibiotic) doses, metabolic oxygen consumption rates are slowed resulting in increased oxygen levels in the sample with respect to antibiotic concentration.^16^ This approach allows for the determination of a minimal biofilm inhibitory concentration (MBIC).^20^ When tested in *P. aeruginosaPAO1* biofilms with 1 to 250 μg/mL antibiotic concentrations, our nanosensor assay measurement (as a proxy for metabolic rate) decreases with increasing antibiotic concentration as expected (Figure 4). The smaller assay response indicates that oxygen concentration is closer to atmospheric values—meaning that oxygen consumption within the biofilm was lower in samples with higher concentrations of antibiotic as expected.

**Figure 5:**
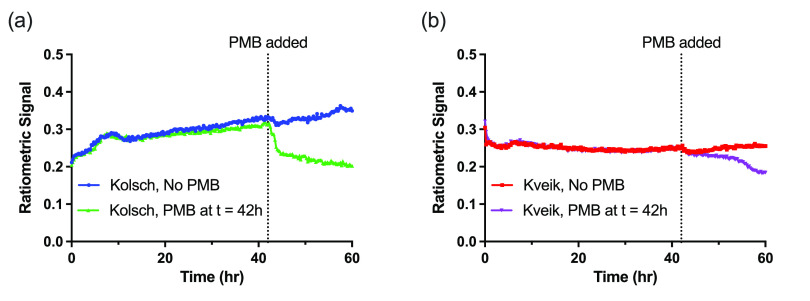
Oxygen consumption in *S. cerevisiae* for (a) Kolsch and (b) Kveik strains was monitored using oxygen nanosensors. Before PMB was added, the oxygen nanosensors continuously monitored oxygen consumption levels in both the Kolsch and Kveik strains. At 42 h after initial incubation, potassium metabisulfite (PMB) was added to inhibit yeast growth and test nanosensor response to yeast oxygen consumption, as indicated by the dotted line. Once PMB was added, the ratiometric signal of the sensors decreased, indicating decreased yeast oxygen consumption in both strains. The Kolsch PMB sample experienced a sharper decrease in oxygen consumption than the Kveik strain, as measured by the ratiometric signal. Error bars have been removed for clarity; figures with error bars can be found in Figures S10–S12 in SI.

The nanosensors were also tested to monitor oxygen consumption in *Saccharomyces cerevisiae* fermentations with potassium metabisulfite (PMB) as an antimicrobial agent to halt metabolic activity—an approach described by Saccomano et al.^21^ As seen in Figure 5, once PMB is added 42 h after yeast incubation, a noticeable decline in the ratiometric signal is observed with both yeast strains, though the Kolsch strain showed a more dramatic initial drop post-PMB addition. The decrease in ratiometric signal indicates a decrease in yeast oxygen consumption and an increase in ambient oxygen concentration indicating the efficacy of the antimicrobial agent as expected. Thus, continuous monitoring of oxygen levels is possible for measuring metabolic activity with these nanosensors. Variations between the two oxygen consumption curves in Figure 5 match previous oxygen consumption trends between the two yeast strains as previously observed.^21^ Thus, the nanosensors can continuously monitor oxygen consumption with adequate reversibility capabilities.

## Discussion

The fabrication of nanosensors with the FNP method provides new potential for the sensing field by coupling previously characterized sensing mechanisms with the scalability and repeatability of FNP nanoparticles. Oxygen sensing was used for testing, as it has been well characterized with other nanosensor formulation methods, and it has applications in tracking metabolic activity from various organisms, including*P. aeruginosa*and*S. cerevisiae* (brewing yeast).^16,21,40^ Because FNP has applications in scalable manufacturing, FNP can be used to improve the scalability and replicability of nanosensors for more widespread diagnostic and exploratory applications.

The nanoparticle self-assembly step requires an amphiphilic block copolymer to stabilize the nanoparticles. We have demonstrated that a range of block copolymers can be used.^3,41^ For this application we used the polystyrene-*b*-polyethylene glycol (PS-*b*-PEG). The reason for this is that the block copolymer is not degradable, and the nanoparticles are stable in aqueous solution for indefinite periods of time. If this sensor technology were translated into clinical applications, then the same nanoparticles could be made from the block copolymer poly(lactic acid)-*b*-polyethylene glycol (PLA-*b*-PEG), which is approved for parenteral drug administration. The nanoparticles made with the same internal components as described in this paper can be made using PLA-*b*-PEG as are made using PS-*b*-PEG. We demonstrated this in the paper by Pagels.^3^

The FNP sensors showed that they have several advantageous properties over traditional nanoparticle synthesis methods such as emulsification solvent evaporation (ESE). The ability to tune the size of the nanoparticles was demonstrated by altering the ratio of the core and structural components. It is possible that we could achieve a wider range of nanoparticle sizes by further optimization of the synthesis composition. Additionally, the sensors showed better stability and similar levels of leaching compared their ESE counterparts with the same dye loading ratio making it more practical to scale and store FNP sensor batches long-term. FNP also demonstrated a higher loading because the ESE tended to form more dye-containing aggregates which get filtered out in the sensor processing protocol.

When the sensors were tested for oxygen responsiveness, the signal of the oxygen-responsive dye (PtTPP) decreased with increasing oxygen concentrations, allowing for a relationship to be drawn between PtTPP signal intensity and oxygen concentration. However, the reference dye (DiA) signal also exhibited slight changes before normalization, decreasing in luminescence with increasing oxygen concentrations, although with a different slope. Fortunately, the normalized pseudo-Stern–Volmer plot (Figure 3) shows that the ratiometric measurement can still be used to correlate luminescence to dissolved oxygen even as the DiA signal is changing, highlighting the value of ratiometric measurements. While the fluctuations in reference dye signal provide an impetus to investigate other reference dye candidates for future formulations, ratiometric changes between the oxygen-responsive dye and reference dye used provide initial indications that the sensors are capable of effectively responding to changes in oxygen concentrations at least 40 days after fabrication and with recalibration up to 100 days after fabrication. Thus, FNP nanosensors can be used for longer term sensing applications.

We also found that the FNP sensors exhibited reversibility when purged with 0% and 21% oxygen environments. However, a gradual decrease in reference dye (DiA) signal led to an increasing luminescence ratio over multiple cycles. The excitation LED was powered for no longer than 5 s per reading, which provided enough time to generate the reversibility emission spectra. Thus, decreasing DiA signals may be attributed to the degradation of DiA with prolonged excitation light exposure over many cycles. Additional testing would be required to determine the effect on full-range oxygen sensing (0 to 6.65 mg/L) that age would contribute to.

Furthermore, determining oxygen concentration in biological samples is of interest, particularly as nanosensors fabricated with alternate approaches functioned in bacterial biofilms and yeast cultures. Preliminary testing with *P. aeruginosa* biofilms indicated that the FNP sensors are capable of sensing oxygen changes in biofilms in response to antibiotic administration. The sensors were also capable of measuring distinct, real-time changes in metabolic activity between different *S. cerevisiae* brewing strains (Kolsch and Kveik) after antibiotic exposure.

In both cases, the decreasing sensor signal with increased antibiotic concentration is indicative of decreasing oxygen utilization caused by cell death, where the fluorescence ratio at high antibiotic doses (where we expect no metabolism) matches that of our atmospheric calibration—implying no metabolic consumption of oxygen. The trends in sensor response that we have observed in these experiments generally match other oxygen sensor studies that have been done previously in similar microbial environments showing a lack of significant change in function of these nanosensors due to the FNP fabrication process. A future application may include investigating the ability of FNP nanosensors to provide a platform for 3D luminescence imaging of biofilms as previously done with PtTFPP/DiA sensors fabricated by an emulsification–solvent evaporation technique.^16^

Our oxygen sensors show that FNP is a viable method for fabricating nanoparticles for analyte measurements. The FNP method for developing nanosensors does not impede the use of dyes of interest for sensing, providing potential for adaptations of existing sensor dyes to measure various other analytes in biological samples.

As nanosensors are developed in the future for more strictly controlled assays, the ability to scale up and produce a consistent product will become very important. Even in exploratory bench-scale experiments, the need for lower batch to batch variabilities makes FNP a desirable method for nanosensor fabrication. Current nanoparticle synthesis methods produce several milliliters of nanosensor solution per batch,^14,39^ where FNP methods could potentially produce liter scale batches of sensor for high-throughput or large volume testing.^1^ The ability to tune nanoparticle size enables better control over parameters such as sensor response time and diffusive properties *in vivo*.^42,43^ Given the ability to implement oxygen sensing into FNP products, future exploration of applicability to ionophore sensors could allow for ion-sensing in various environments.

## Conclusion

FNP is a valuable technique that enables fast and reliable fabrication of nanosensors. When a typical nanoprecipitation formulation of oxygen nanosensors was fabricated with the FNP process, the sensors exhibited sensing behavior that was similar to other fabrication methods, allowing for the sensor components to function as originally intended. The nanosensors function in biological systems, measuring the antibiotic and antimicrobial agent mediated changes in *P. aeruginosa* and *S. cerevisiae* metabolism. In all of the tested conditions, the sensors were able to measure oxygen in real time with adequate reversibility, as FNP allows the existing sensor mechanisms to work effectively. Nanosensor technology can benefit from the uniformity of FNP products to ensure uniform population for a given application, and while FNP had not been previously applied to nanosensors, this work opens up possibilities for future FNP-based nanosensors for various analytes. Future applications can include adaptations to other oxygen nanosensor formulations, ion sensors, and testing in other biological systems.

## Supporting Information Available

Figure S1 – Sample selection: nanoparticle size under various synthesis conditions. Figure S2 – Sample selection: nanoparticle size distribution under various synthesis conditions. Figure S3 – Sample selection: raw luminescence spectra under various synthesis conditions at 420 nm excitation. Figure S4 – Sample selection: raw luminescence spectra under various synthesis conditions at 650 nm emission. Figure S5 – Nanosensor encapsulation and dye leaching for FNP and ESE methods. Figure S6 – Aggregation-Caused Quenching: dye dissolved in particle vs organic solvent. Figure S7 – Batch nanoparticle size, polydispersity, zeta potential and mobility for FNP vs ESE fabrication methods. Figure S8 – Nanoparticle size and polydispersity changes over time. Figure S9 – Raw and normalized luminescence spectra variations for PtTPP/DiA sensors at different oxygen concentrations. Figure S10 – Stern–Volmer (SV) Plots for Individual Dyes as Compared to Ratiometric Signal. Figure S11 – Luminescence intensity and ratiometric response of DiA and PtTPP showing nanosensor reversibility. Figure S12 – Raw luminescence spectra of nanosensor response at 40 days and 100 days after production. Figure S13 – Raw luminescence spectra of nanosensor response to pH 5–8 solutions. Figure S14 – Antibiotic Susceptibility Testing (AST) with *P. aeruginosa*: Raw signal of dyes during first 5 h. Figure S15 – AST data with *P. aeruginosa*: normalized data of Figure S8. Figure S16 – Yeast metabolic activity assay: ratiometric signal data with error bars. Figure S17 – Yeast metabolic activity assay: individual dye signal data with error bars. Figure S18 – Yeast metabolic activity assay: no nanosensor and no yeast controls with error bars. (PDF)

## Abbreviations

FNP: Flash Nanoprecipitation
PtTPP: Platinum(II) 5,10,15,20-(tetraphenyl)porphyrin
DiA: 4-(4-Dihexadecylaminostyryl)-*N*-methylpyridinium iodide
PS–PEG: polystyrene-*block*-polyethylene glycol
