# Supplementary material for: Sensors in a Flash! Oxygen Nanosensors for Microbial Metabolic Monitoring Synthesized by Flash Nanoprecipitation
Source: ACS Sens. 2022 Sep 2;7(9):2606–14. doi: 10.1021/acssensors.2c00859 (PMC9513798; doi:10.1021/acssensors.2c00859)

Supplementary Information

"Sensors in a Flash! Oxygen nanosensors for microbial metabolic monitoring synthesized by Flash Nanoprecipitation"

Tony Tien[1], Samuel C. Saccomano[1], Pilar A. Martin[1], Madeleine S. Armstrong[2], Robert K. Prud'homme[2], Kevin J. Cash[1,3]*

[1]Chemical and Biological Engineering, Colorado School of Mines, Golden, CO 80401, USA
[2]Chemical and Biological Engineering, Princeton University, Princeton, NJ 08544, USA
[3]Quantitative Biosciences and Engineering, Colorado School of Mines, Golden, CO 80401, USA
* kcash@mines.edu

Figure S1 Sample selection: nanoparticle size under various synthesis conditions

Figure S2 Sample selection: nanoparticle size distribution under various synthesis conditions

Figure S3 Sample selection: raw luminescence spectra under various synthesis conditions at 420 nm excitation

Figure S4 Sample selection: raw luminescence spectra under various synthesis conditions at 650 nm emission

Figure S5 Nanosensor encapsulation and dye leaching for FNP and ESE methods

Figure S6 Aggregation-Cause Quenching: dye dissolved in particle vs organic solvent

Figure S7 Batch nanoparticle size, polydispersity, zeta potential and mobility for FNP vs ESE fabrication methods

Figure S8 Nanoparticle size and polydispersity changes over time

Figure S9 Raw and normalized luminescence spectra variations for PtTPP/DiA sensors at different oxygen concentrations

Figure S10 Stern-Volmer (SV) Plots for Individual Dyes as Compared to Ratiometric Signal

Figure S11 Luminescence intensity and ratiometric response of DiA and PtTPP showing nanosensor reversibility

Figure S12 Raw luminescence spectra of nanosensor response at 40 days and 100 days after production

Figure S13 Raw luminescence spectra of nanosensor response to pH 5-8 solutions

Figure S14 Antibiotic Susceptibility Testing (AST) with *P. aeruginosa*: Raw signal of dyes during first 5 hours

Figure S15 AST data with *P. aeruginosa*: normalized data of Figure S8

Figure S16 Yeast metabolic activity assay: ratiometric signal data with error bars

Figure S17 Yeast metabolic activity assay: individual dye signal data with error bars

Figure S18 Yeast metabolic activity assay: no nanosensor and no yeast controls with error bars

Figure S1. Different ratios of PS-PEG polymeric shell material and Vitamin E or Vitamin E acetate core material show varying average nanoparticle size as measured by Dynamic Light Scattering. Samples with a higher amount of PS-PEG clearly correlated to a smaller nanoparticle size most likely due to the kinetic of nanoparticle stabilization being faster compared to the nucleation of the core. For the core materials, the inverse correlation seemed to true where greater amounts of core material lead to greater particle size. Vitamin E seemed to show a slightly greater nanoparticle size compared to Vitamin E acetate samples containing the same amount of core and shell materials. The dye ratio did not seem to affect the nanoparticle size significantly.

Sample	Dye Mass Ratio (mg PtTPP: mg DiA)	PS-PEG (mg/mL)	Core Material	Core Loading (mg/mL)	NP size (nm)
1	5:0.2	5	Vit E acetate	5	70
2	5:0.2	1.25	Vit E acetate	5	110
3	5:0.2	5	Vit E	5	83
4	5:0.2	1.25	Vit E	5	116
5	5:2	5	Vit E acetate	5	66
6	5:2	1.25	Vit E acetate	5	109
7	5:0.2	5	Vit E acetate	10	102
8	5:0.2	1.25	Vit E acetate	10	158
9	5:2	5	Vit E acetate	10	125
10	5:2	1.25	Vit E acetate	10	160

Figure S2. The particle size distribution is shown for each of the 10 samples correlating those listed in Table 1 and Figure SX. Almost all of the samples show a single peak distributed around the average peak size with exceptions of sample 6 and 9 showing small peaks at the 1 micron scale.

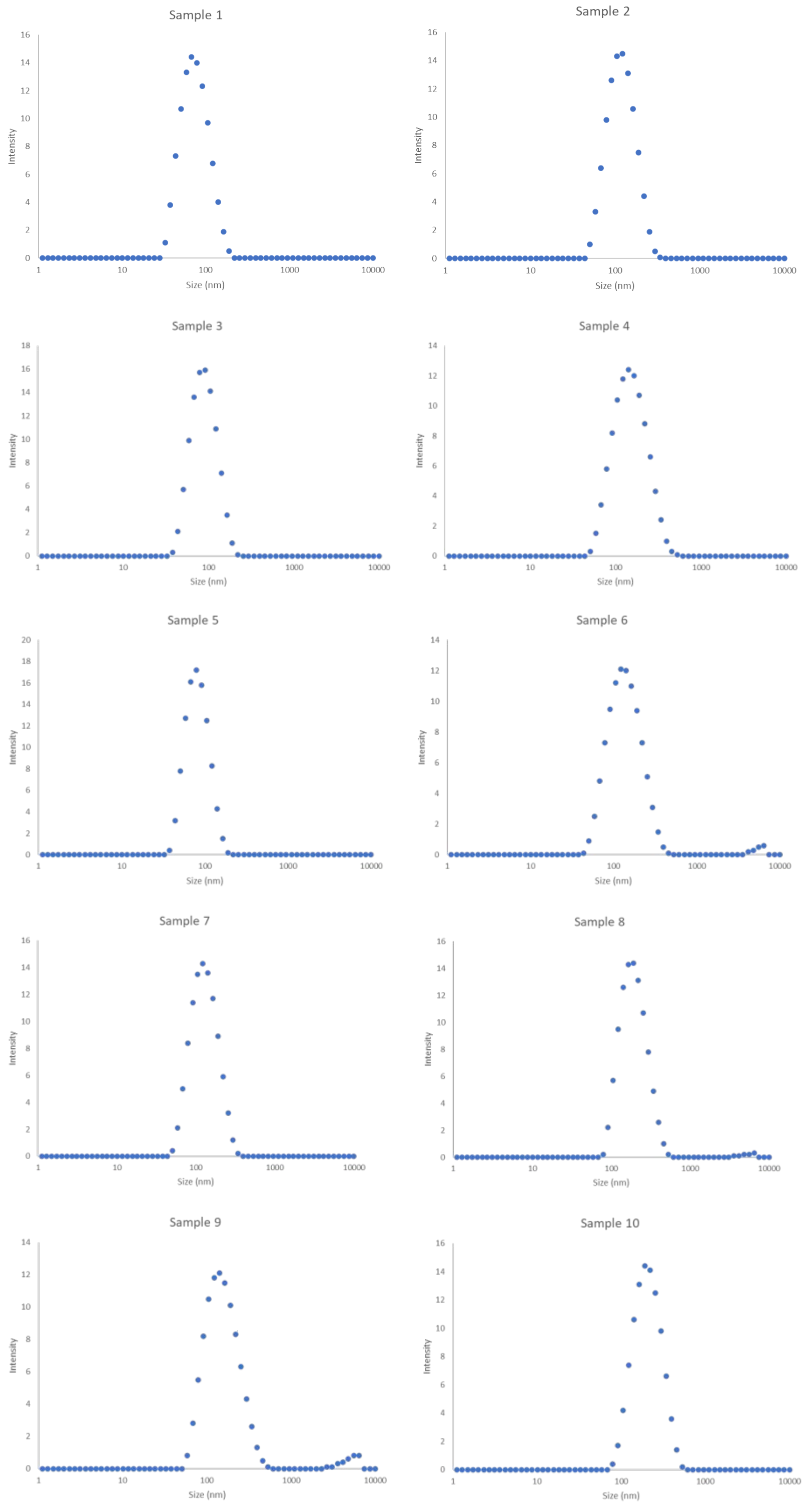

Figure S3. Raw luminescence of ten different formulations of the FNP oxygen nanosensors were tested while excited at 420 nm. In tandem with **Figure S2**, Sample 5 was selected for further testing in this work as it exhibited the best contrast between the deoxygenated and oxygenated conditions in both spectral tests.

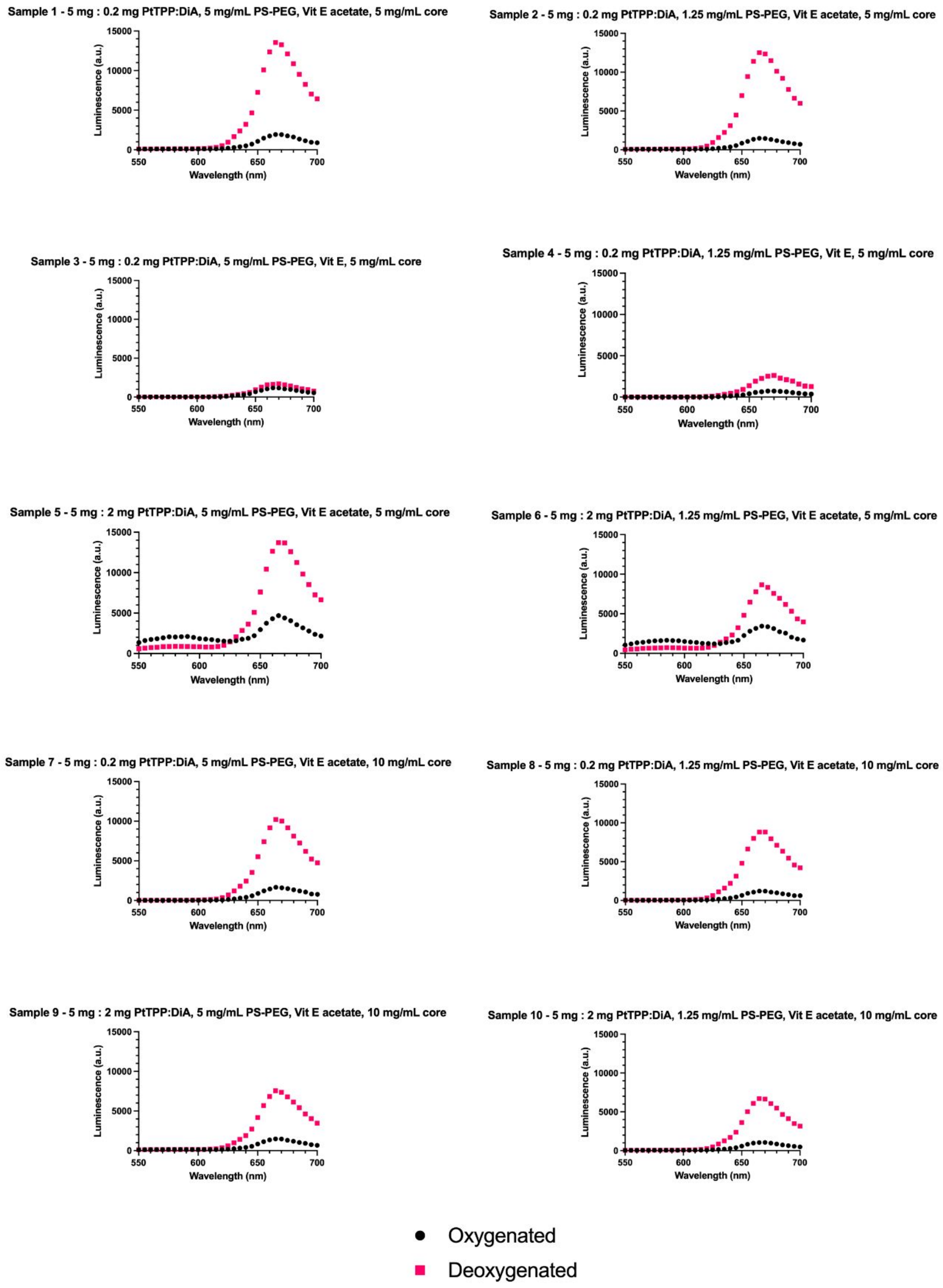

Figure S4. Raw excitation spectra of ten different formulations of the FNP oxygen nanosensors were tested for peak luminescence at 650 nm emission. In tandem with **Figure S1**, Sample 5 was selected for further testing in this work as it exhibited the best contrast between the deoxygenated and oxygenated conditions in both spectral tests.

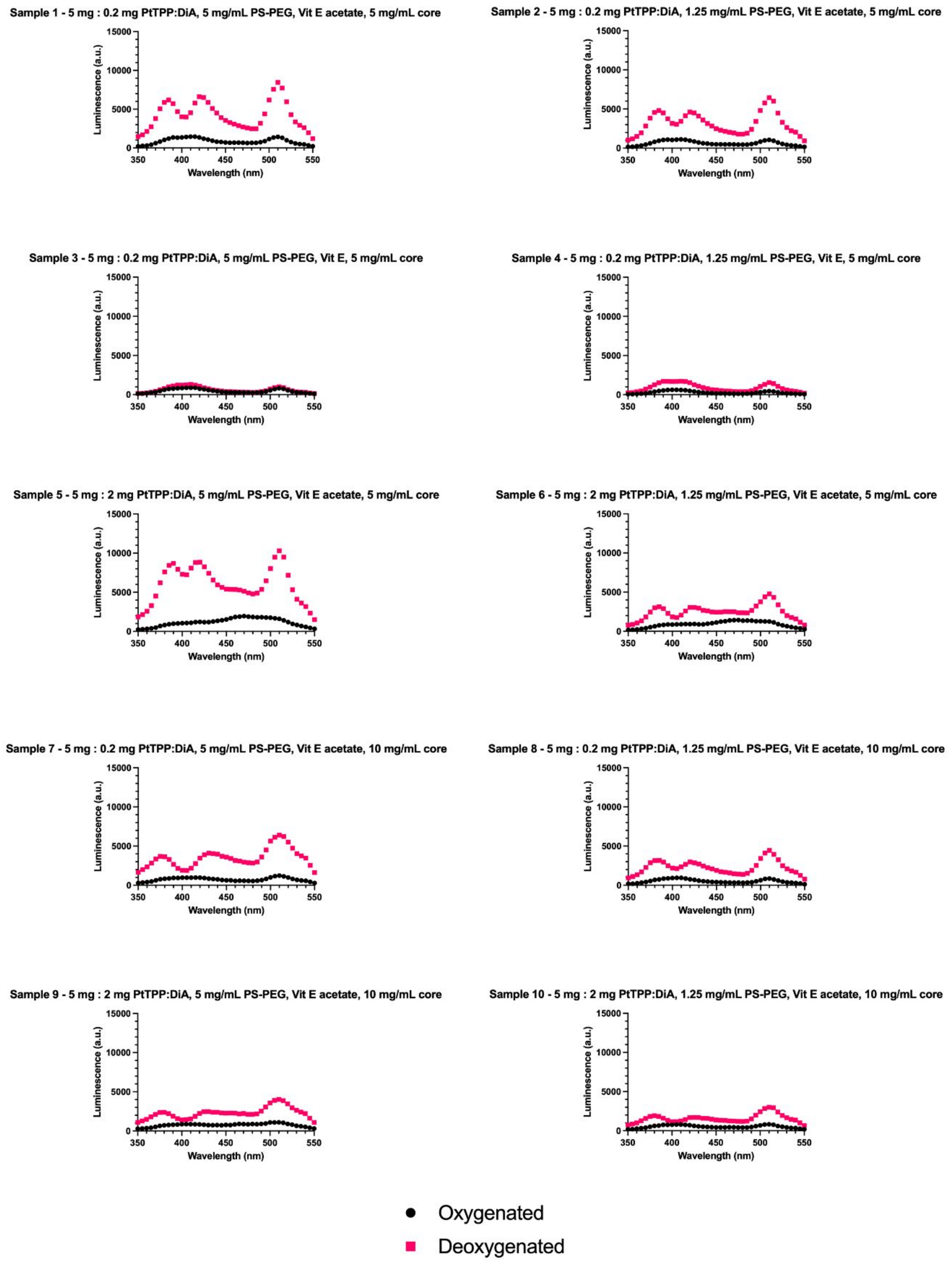

Figure S5. Encapsulation of the PtTPP and DiA dyes was compared for the FNP and SESE samples by measuring the absorbance of the dye peaks of the fresh nanosensor sample (n=3). Each batch of sensors was loaded with the same mass of each dye (125 µg PtTPP, 50 µg DiA). Additionally, the sensors were filtered based on molecular weight to compare the absorbance of (a) particle loaded dye (retentate) and (b) dye in solution (filtrate). The FNP formulation shows a greater signal in the retentate, while neither dye showed a significant peak in the filtrate. This likely means that the dye loading was close to 100% for the FNP method, while the SESE method was much lower, however the excess dye cannot be seen because it was likely filtered out during post-processing steps. (c-f) The absorbance was tracked over 2 weeks to observe the kinetics of dye leaching for each method for PtTPP and DiA in the retentate (c,e) and filtrate (d,f). The FNP sensors showed no increase in signal in the filtrate implying that there was no leaching while a small amount of signal was observed in the SESE sensors.

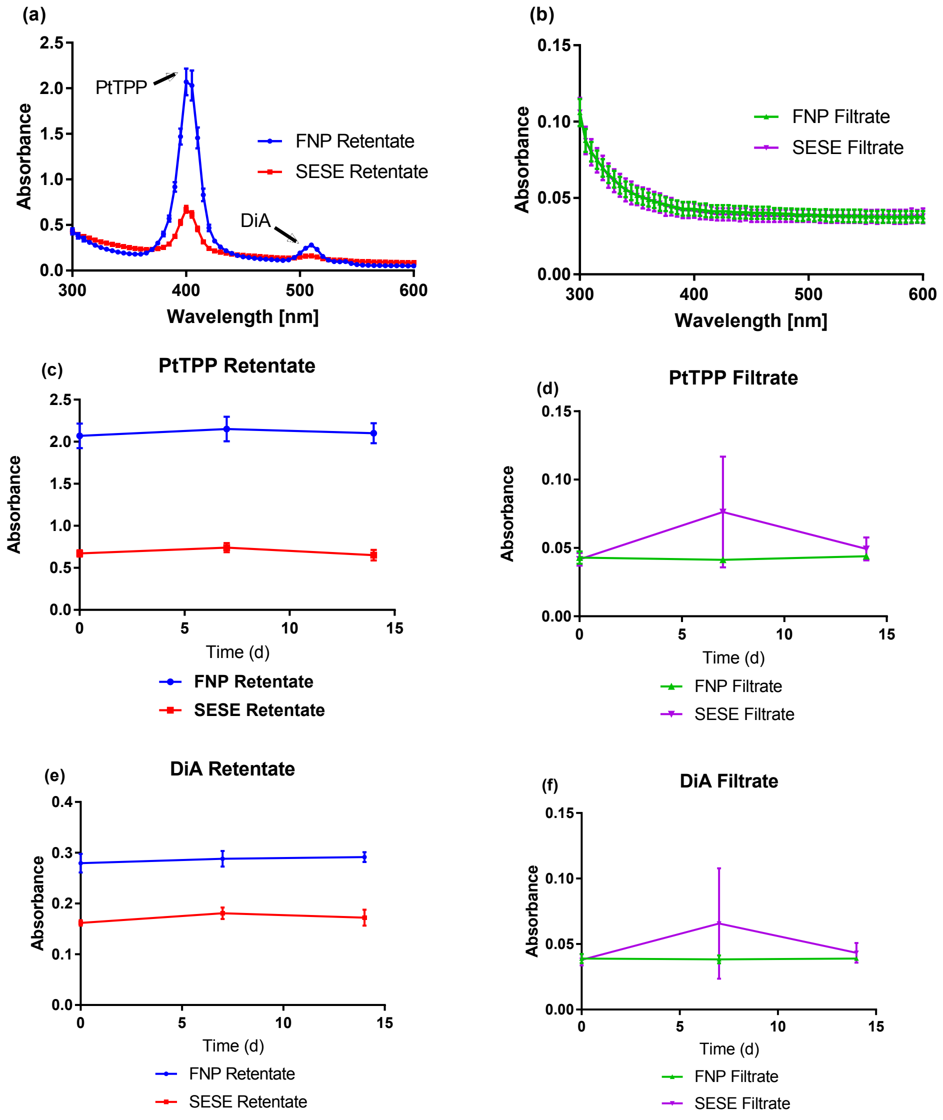

Figure S6. Aggregation-Caused Quenching (ACQ) can occur when organic dyes are encapsulated into nanoparticle matrices reducing the emission signal of the dye compared to when dispersed in solution. An equivalent concentration of PtTPP and DiA as in the nanoparticles was dissolved into dichloromethane to compare fluorescent signal intensity when excited by a 405 nm laser. The dyes show a greater ability to fluoresce in the nanoparticle solution indicating that ACQ is unlikely, as ACQ would typically decrease the fluorescence form the nanoparticle encapsulated dye. Further testing would be needed in order to prove ACQ is not in the system as other factors could be affecting the fluorescence, such as the solvatochromic properties of the dyes, or direct quenching of the dye by the solvent.

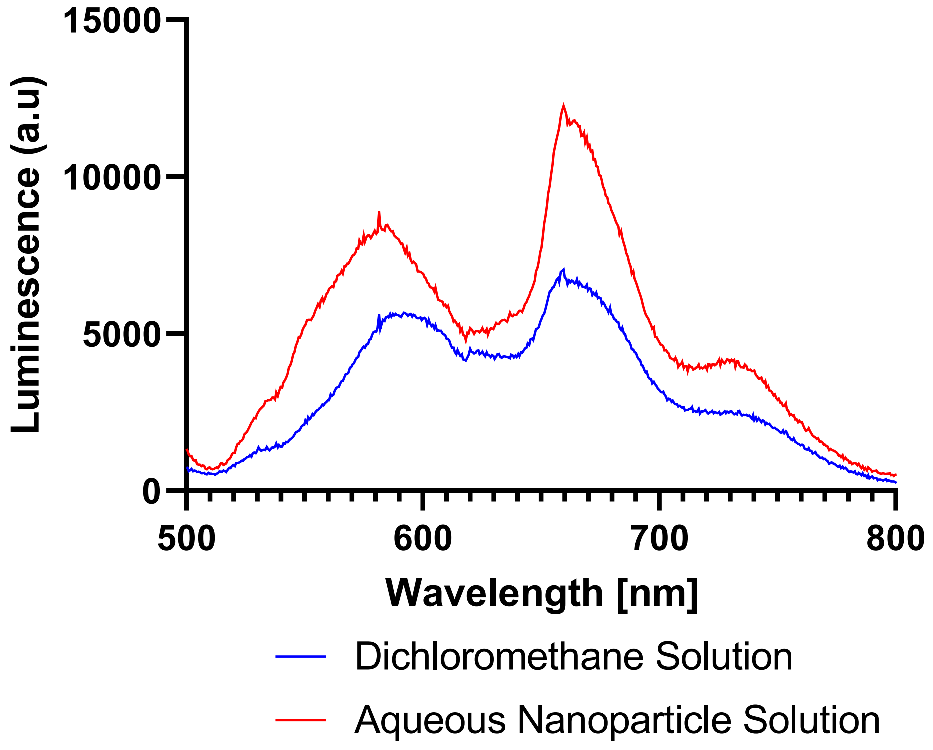

Figure S7. Sample 5 was further analyzed by DLS and ZetaPals for particle size, polydispersity, zeta potential and mobility as compared to samples manufactured by a single emulsion solvent evaporation (SESE) method. The FNP sensor shows a smaller particle size as well as a much narrower polydispersity indicating a sharper distribution of particle diameters. The zeta potential and mobility of the FNP particles was less negative than the SESE particles.

a.) Flash Nanoprecipitation Formulation

Sample	Diameter (nm)	Polydispersity	Zeta Pot (mV)	Mobility
Batch 1	98.27	0.06	-14.43	-1.13
Batch 2	92.31	0.06	-19.95	-1.56
Batch 3	88.73	0.03	-15.00	-1.17
Average	93.10 ± 3.93	0.05 ± 0.02	-16.46 ± 2.48	-1.29 ± 0.19

b.) Solvent Evaporation Formulation

Sample	Diameter (nm)	Polydispersity	Zeta Pot (mV)	Mobility
Batch 1	181.23	0.17	-47.59	-3.72
Batch 2	168.53	0.19	-54.35	-4.25
Batch 3	178.52	0.17	-45.98	-3.59
Average	176.09 ± 5.46	0.018 ± 0.01	-49.31 ± 3.62	-3.85 ± 0.29

Figure S8. Nanoparticle size and polydispersity was measured over a two week time period (n=3) to assess the size-related stability of the particle formulation. Both the FNP and ESE formulations seem to show a minimal change in particle over the time course though some variability in the polydispersity was seen. Both FNP and ESE samples showed a greater polydispersity at the 14 day time point though we believe one of our data points to be an outlier as the calculate Grubb's Test value was 1.38, which is greater than the allowed value of 1.15 for data sets with n=3. Overall the stability of the particles does not seem to be a concern after 14 days from the batch synthesis.

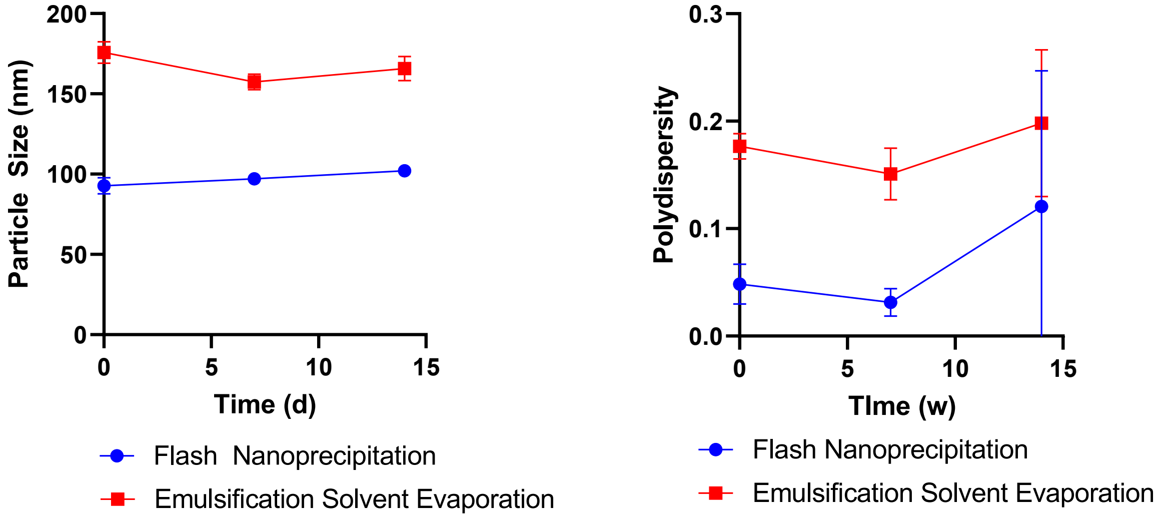

Figure S9. Variations of **Figure 2** from the main text are provided here with additional detail. Provided are (a) normalized spectra with error bars, (b) raw spectra without error bars, and (c) raw spectra with error bars. Graphs without error bars are provided solely for ease of visualization. In all variations, PtTPP signal at the dotted line labeled “PtTPP” decreases in accordance with increasing oxygen concentrations. In raw spectra graphs (b) and (c), some fluctuation in DiA luminescence occurs at the dotted line labeled “DiA”, but because DiA is used as a reference dye, ratiometric comparison with PtTPP is still possible to measure oxygen concentrations.

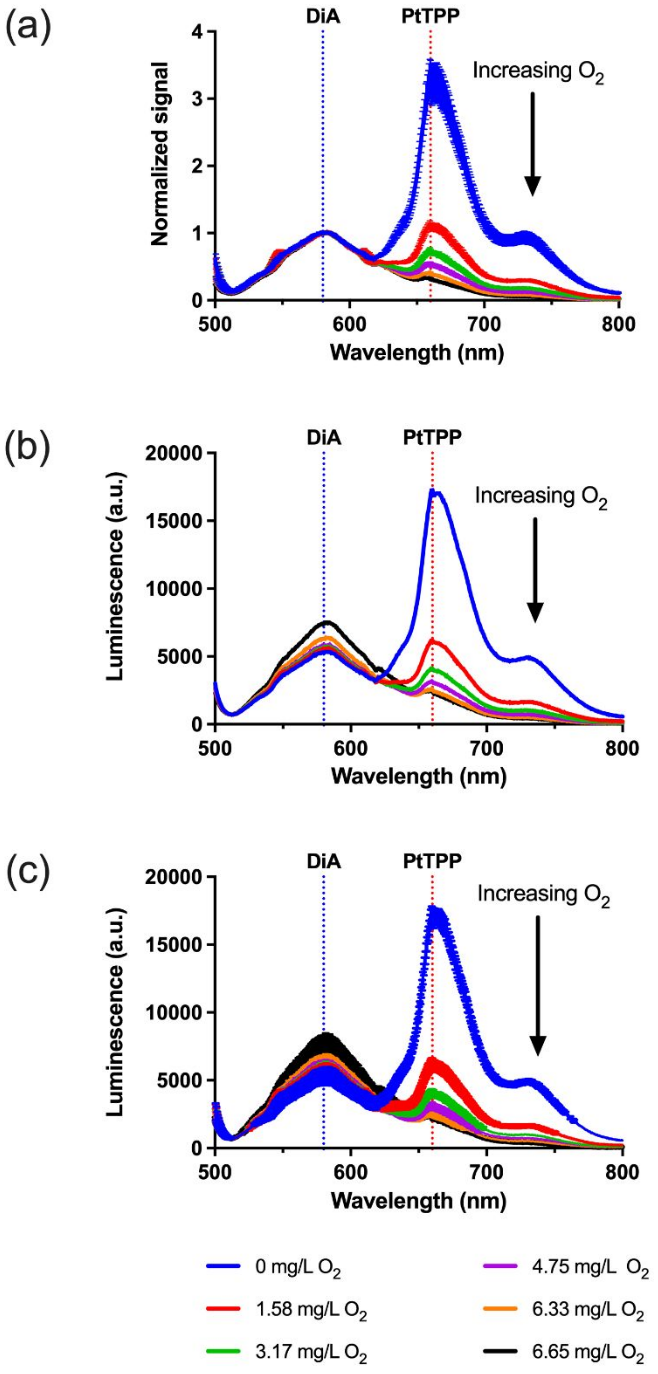

Figure S10. The Stern-Volmer plot shows the response of the two dyes to oxygen as compared to the ratiometric pseudo-Stern Volmer plot shown in **Figure 3**. PtTPP exhibits a relatively linear change in luminescence as dissolved oxygen concentration increases. DiA luminescence remains relatively constant, allowing for optimal ratiometric comparison. The provided equation is the same as **Equation 1** in the main text with the exception that the luminescence of the individual dyes – not the ratio of the two – are used for the respective DiA and PtTPP points and fits. * indicates the dissolved oxygen concentration estimated from a 21% oxygen concentration in the gas mixture.

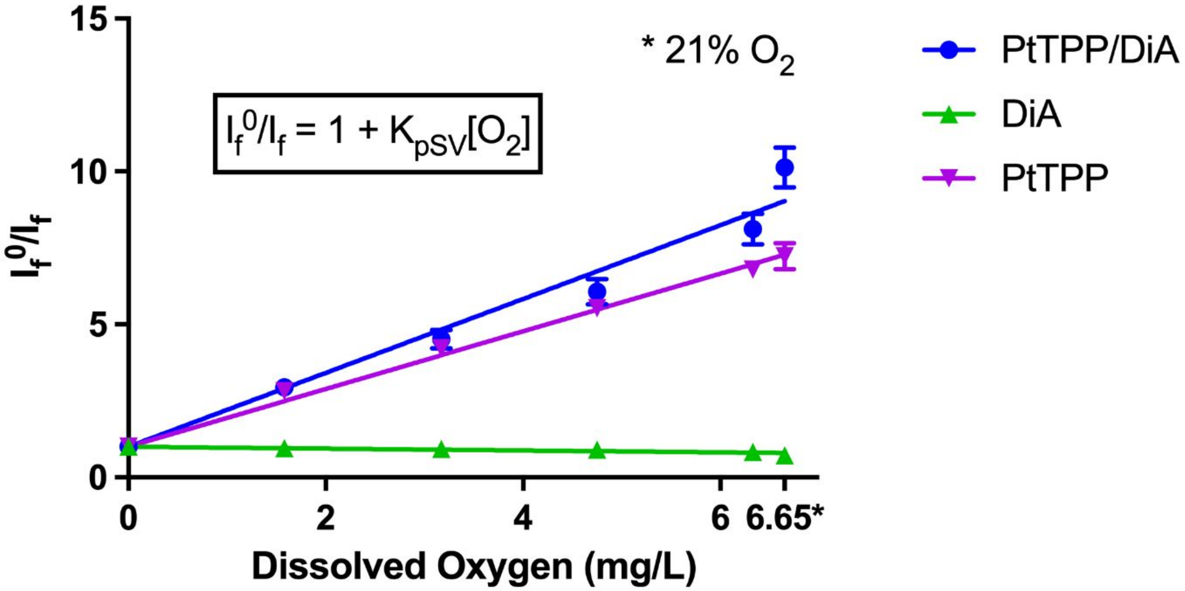

Figure S11. Reversibility tests with gas bubbling were performed to ensure the ability of the sensors to respond in real time according to local oxygen concentrations. In the graphs below, each cycle represents full oxygenation of the system (21% O_2) , followed by full deoxygenation of the system (0% O_2). Provided are luminescence data for (a) DiA, (b) PtTPP, and (c) ratiometric signal (PtTPP/DiA). DiA exhibited a gradual decrease in luminescence over multiple cycles, as seen in (a), but its use as a reference dye still allows for ratiometric analysis of oxygen concentrations. PtTPP responds in a relatively consistent fashion at 0% O_2 and 21% O_2 levels, as seen in (b).

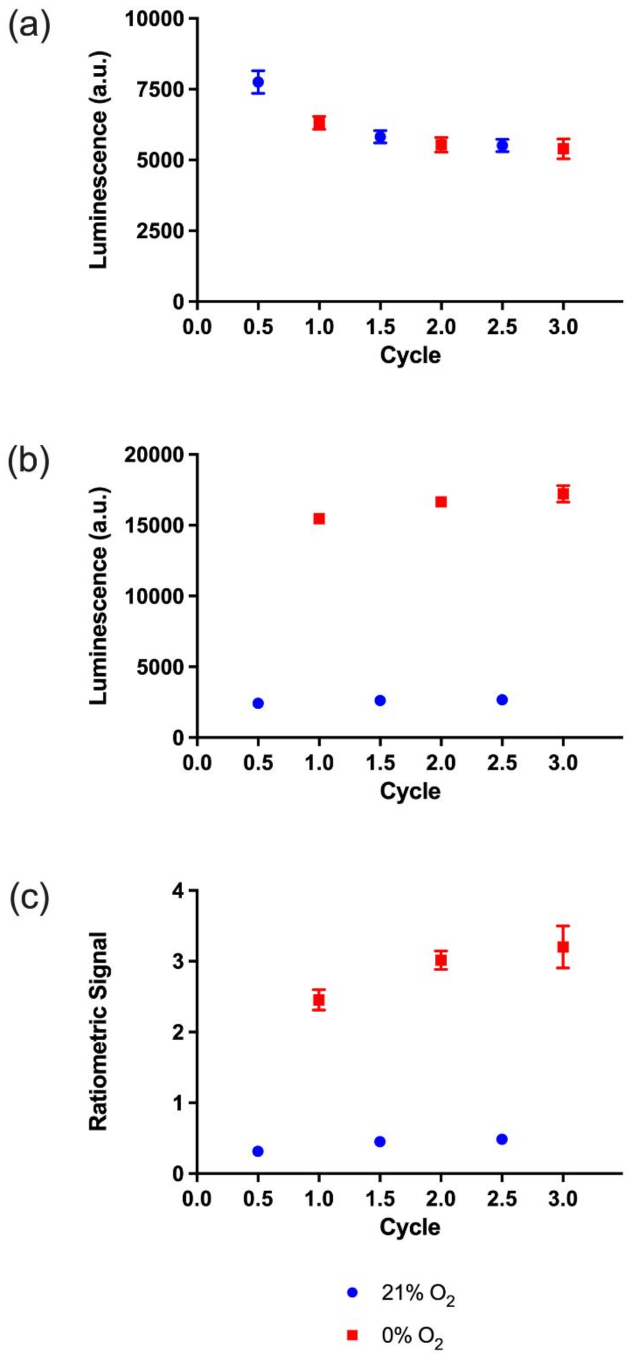

Figure S12. The brightness of the sensors was tested at (a) 40 days after production and (b) 100 days after production to determine the lifetime. While the luminescence of the dyes decreases over time, recalibration based on DiA's presence as a reference dye allows for continued use of the sensors for oxygen sensing.

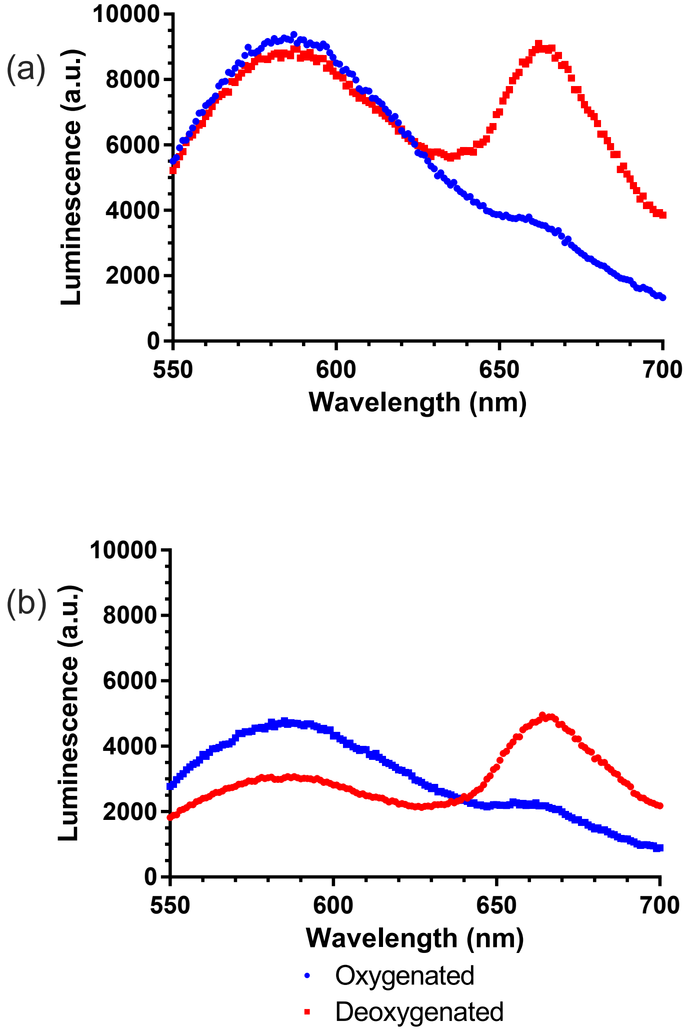

Figure S13. The response of the sensors to pH was tested in a biologically relevant range of pH 5-8 using HCl and NaOH to adjust the pH of the buffer solution. At 488 nm excitation, the DiA peak at 580 nm exhibits minimal change in signal regardless of pH. The PtTPP peak at 660 nm is not visible in these graphs as these tests were performed at ambient oxygen conditions (6.65 mg/L) at 5675 ft elevation. Provided is (a) the full spectra and (b) the same spectra but zoomed in from 560-620 nm for visualization.

(a)

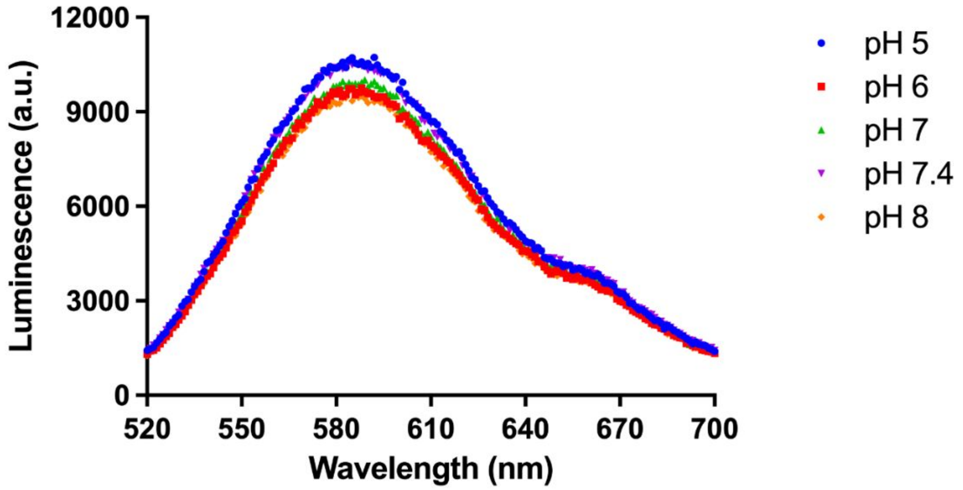

(b)

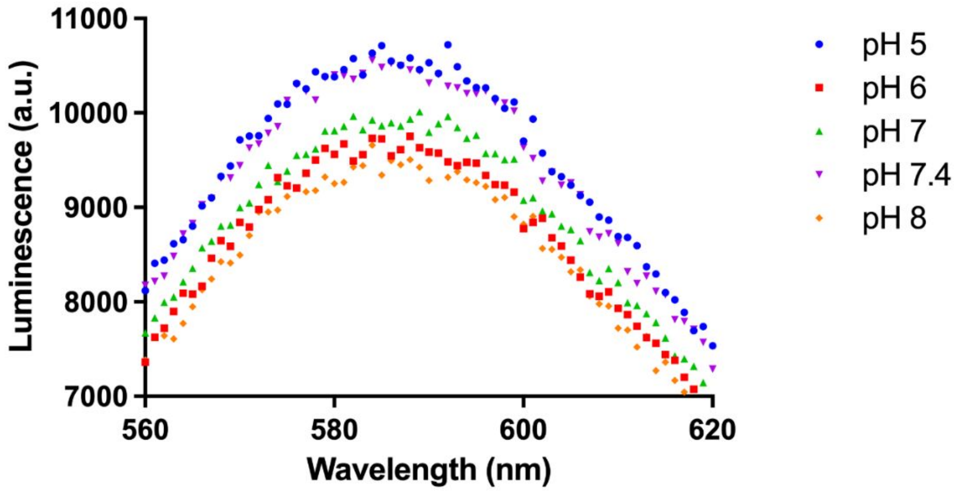

Figure S14. The raw luminescence data for (a) DiA at 580 nm and (b) PtTPP at 660 nm are provided below over the 5-hour growth period used for Assay Response determination in **Figure 4**. Linear regressions were calculated over the 5-hour time period for each condition and dye, and the ratiometric comparison of the two provides the Assay Response.

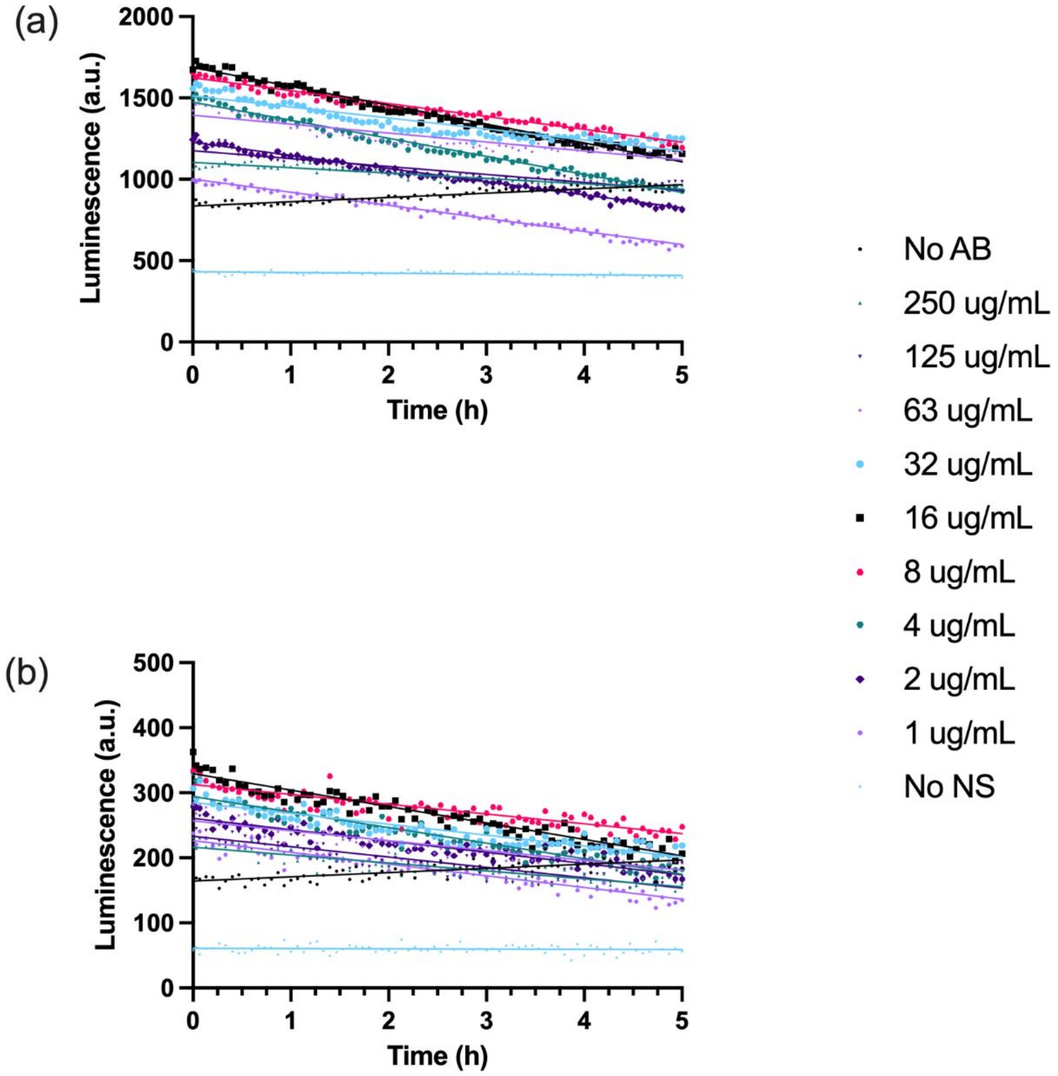

Figure S15. Ratiometric comparison of the data shown in **Figure S8** yields (a) the luminescence ratio data and (b) normalized luminescence ratio data. The normalization of (a) to (b) was performed by dividing the luminescence ratios at each time point by the initial luminescence ratio at time = 0 h for the respective conditions.

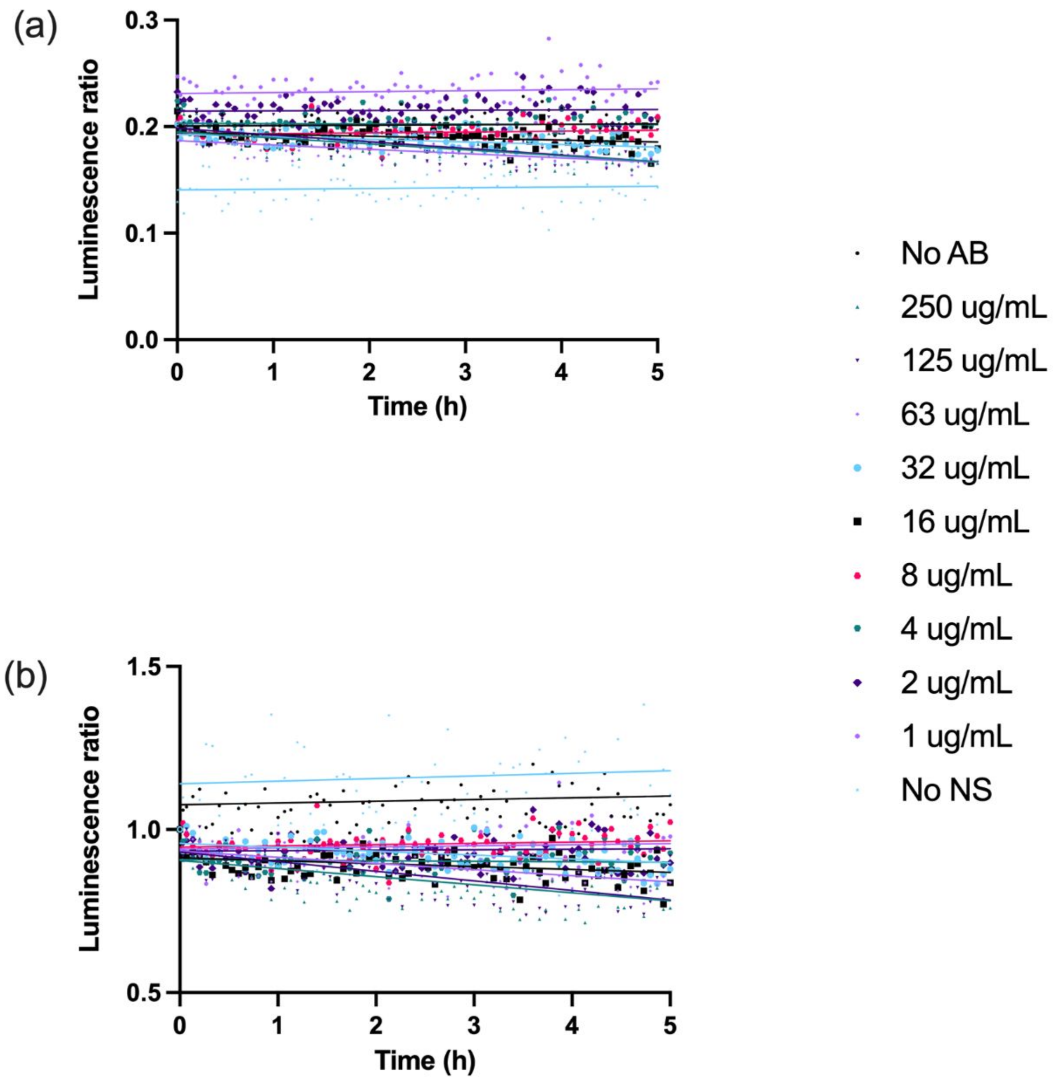

Figure S16. The graphs provided in **Figure 5** are provided here with error bars for (a) Kolsch and (b) Kveik. The range of error was relatively consistent for both PMB and no PMB conditions for both yeast strains. While the distinction between the PMB and no PMB conditions are more distinct after PMB is added (dotted line) for (a) Kolsch than (b) Kveik, a notable difference between the two conditions still occurs in both yeast strains.

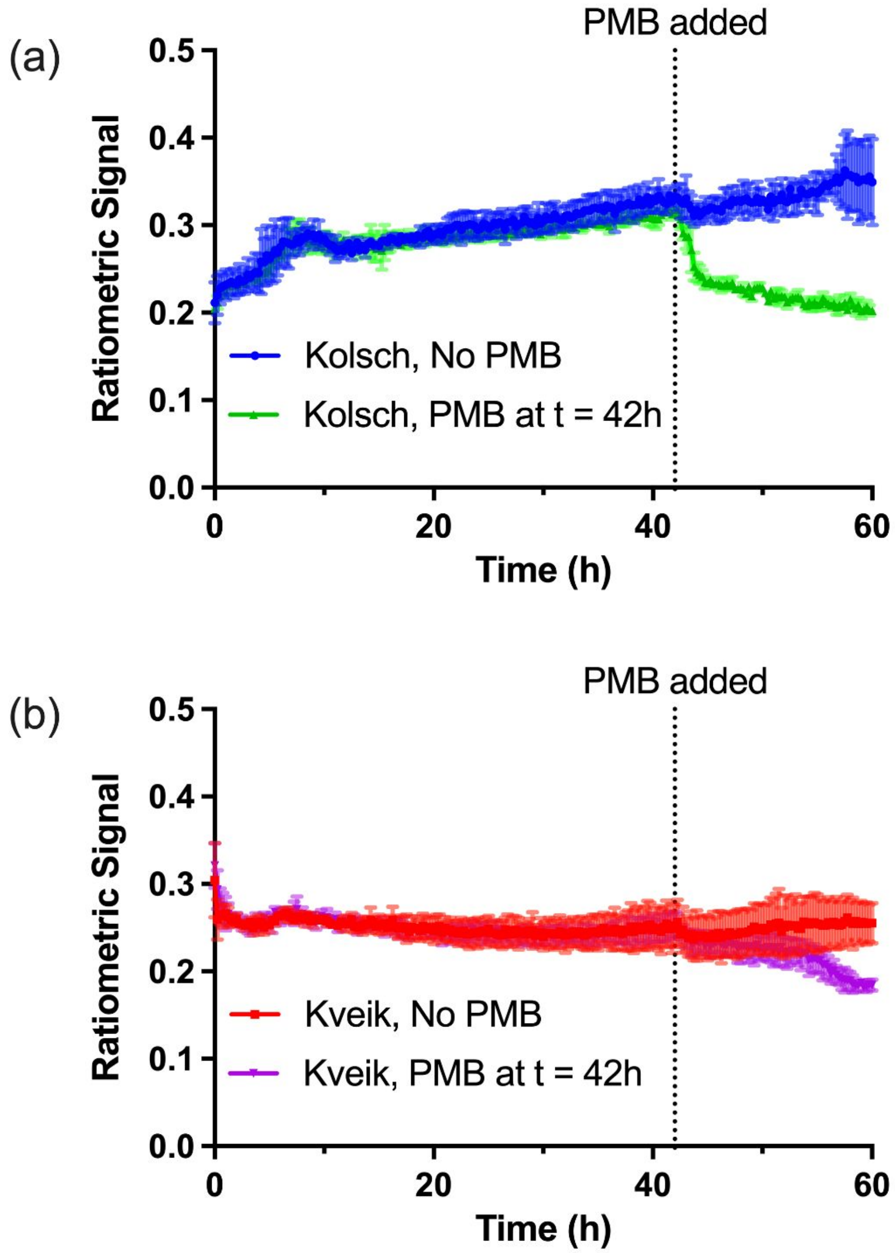

Figure S17. The individual dye data for (a) DiA at 580 nm and (b) PtTPP at 660 nm are displayed over the course of the assay. The ratiometric data of (b) over (a) (PtTPP/DiA) was used to generate the ratiometric data shown in **Figure 5** and **Figure S10**. Consistent changes in the NS Only and NS + PMB conditions ensure consistency of the sensors over time.

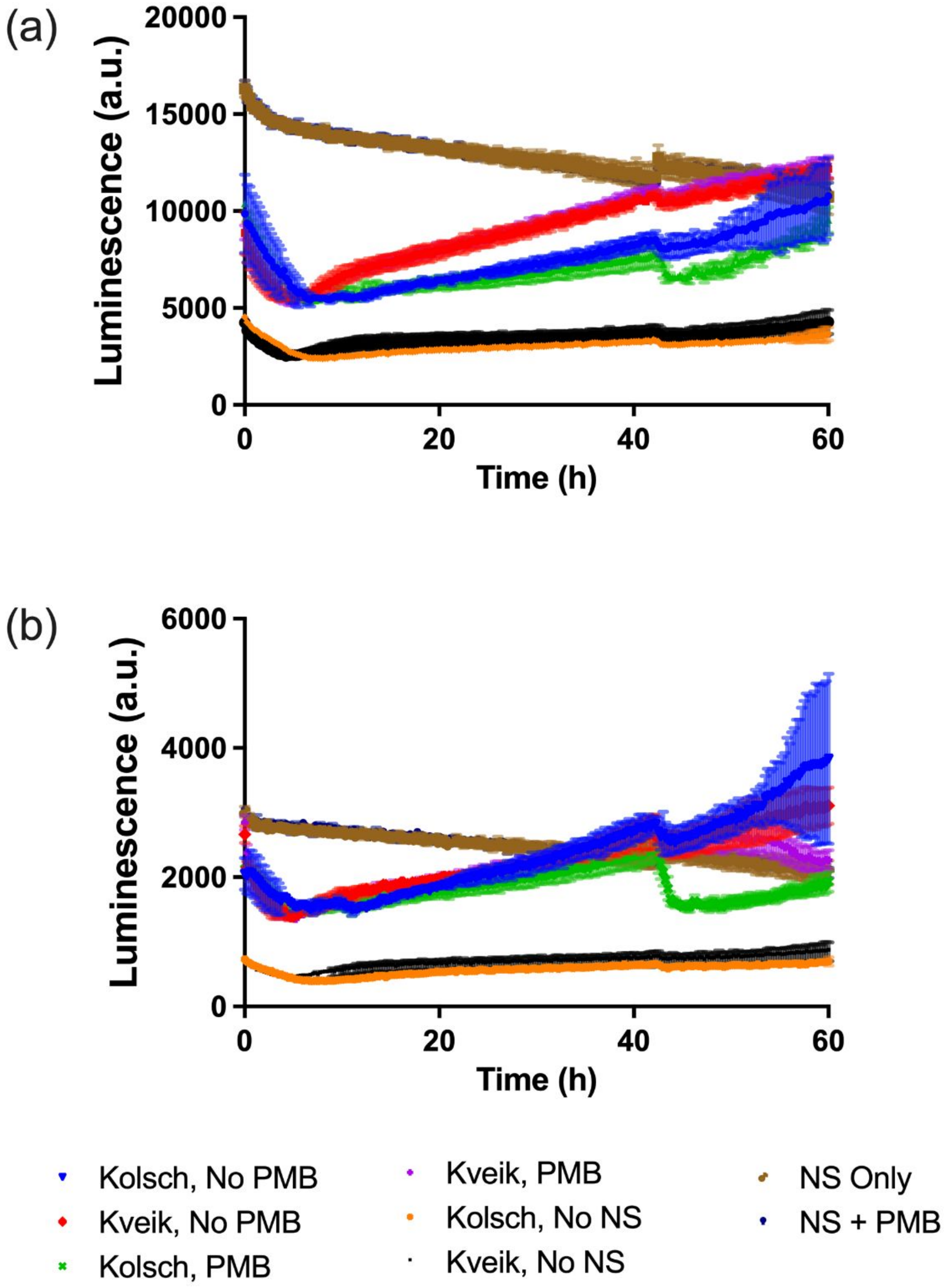

Figure S18. The graph provided below provides a comparison of the yeast control conditions without nanosensors in comparison with nanosensor interactions with and without potassium metabisulfite (PMB). The nanosensors respond relatively consistently regardless of the addition of PMB, allowing for them to be used for the assay.

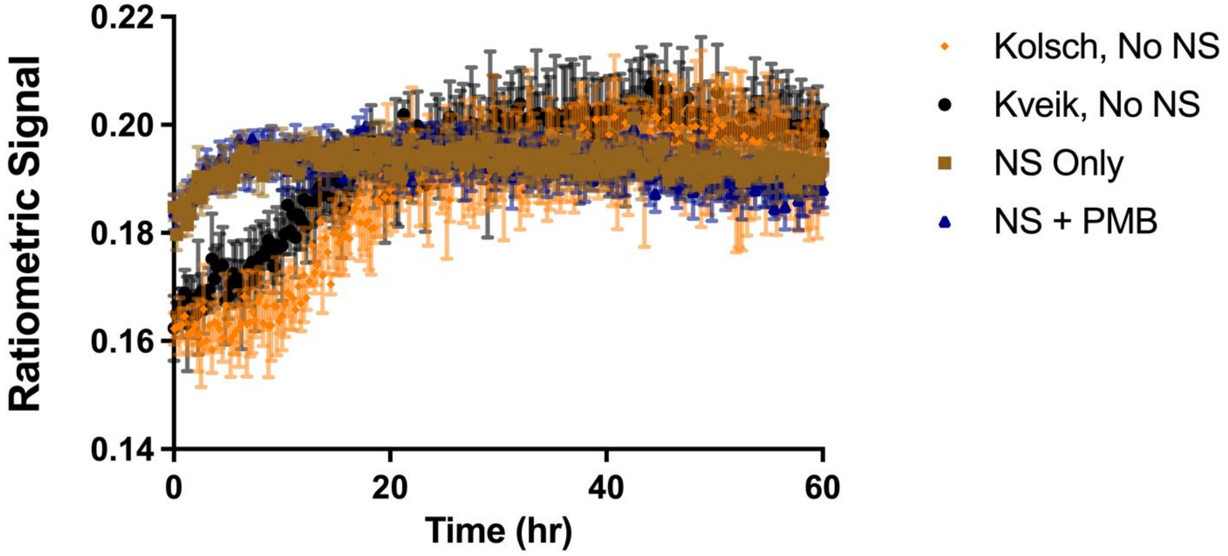

Supplement: Supplementary file 1 — se2c00859_si_001.pdf [file se2c00859_si_001.pdf]
